# Temporal trends in cross-country inequalities of Burkitt lymphoma burden in children under 10 years of age from 1990 to 2021

**DOI:** 10.1097/MD.0000000000048617

**Published:** 2026-05-08

**Authors:** Siqi Zhang, Tian Xia, Yu Li, Bin Pei, Ren Niu, Guoxin Huang, Xiong Wang

**Affiliations:** a Anesthesia Room, Luzhou Maternal and Child Health Hospital (Luzhou Second People’s Hospital), Luzhou, China; b Department of Medical Oncology, Xiang’an Hospital of Xiamen University, School of Medicine, Xiamen University, Xiamen, Fujian, China; c Department of General Surgery, Xiangyang No. 1 People’s Hospital, Hubei University of Medicine, Xiangyang, Hubei, China; d Center of Evidence-Based Medicine, Xiangyang No. 1 People’s Hospital, Hubei University of Medicine, Xiangyang, Hubei, China; e Center of Cell Therapy, Xiangyang No. 1 People’s Hospital, Hubei University of Medicine, Xiangyang, Hubei, China.

**Keywords:** Burkitt lymphoma, disease burden, GBD 2021, trends

## Abstract

Burkitt lymphoma (BL) is a highly aggressive form of non-Hodgkin lymphoma. Given the uncertainty surrounding the exact global incidence rate of BL, this study aims to provide a comprehensive analysis of BL epidemiology in children under 10 years of age at the global, regional, and national levels, along with a multidimensional trend analysis. This study utilizes the standardized methodology from the 2021 Burden of Disease (GBD) to describe the incidence, prevalence, mortality, and disability-adjusted life years (DALYs) of BL in children under 10 years of age at the global, regional and national levels. The analysis includes both numerical counts and age-standardized rates (ASR) per 1,00,000 individuals, along with their 95% uncertainty intervals (UI). The burden of BL is decomposed based on population growth, aging, and epidemiological changes. Additionally, cross-national health inequalities in BL burden are quantified using standard health equity methods, and the burden of BL is projected to 2040. In 2021, there were approximately 22,878.70 (95% UI: 14,619.46–30,361.43) cases of BL in children under 10 years of age globally, with Western and Eastern Sub-Saharan Africa showing the highest incidence, prevalence, mortality and DALYs. Nigeria had the highest number of cases, with approximately 4624.35 (95% UI: 2192.40–7001.90) cases. From 1990 to 2021, the global burden of BL in children under 10 years of age showed an overall upward trend. Decomposition analysis revealed that low Socio-demographic Index (low-SDI) regions saw the most significant increase in DALYs, primarily driven by population growth. Inequality analysis indicated that countries with lower SDI continue to bear a growing burden of BL in children under 10 years of age. Notably, projections suggest that the incidence, prevalence, mortality, and DALYs for BL in children under 10 years of age will continue to rise, with global cases expected to reach 23,755 by 2040, with females exhibiting a relatively faster increase in disease burden compared with males. This study provides a comprehensive analysis of the disease burden of BL in children under 10 years of age, highlighting that regions and countries with lower SDI bear a significantly higher burden. Additionally, cross-national inequalities in BL burden have worsened over time.

## 1. Introduction

Burkitt lymphoma (BL) is a highly aggressive form of non-Hodgkin lymphoma, which can be classified epidemiologically into endemic, sporadic and immunodeficiency-associated types.^[1]^ Endemic BL predominantly occurs in equatorial Africa and is the most common pediatric malignancy in sub-Saharan Africa.^[2]^ It is primarily limited to areas with stable malaria transmission.^[3,4]^ In equatorial Africa, endemic BL accounts for 50% of all childhood cancers, with a notable association with early Epstein–Barr virus (EBV) infection.^[5,6]^ This type mainly affects children aged 4 to 7, often presenting with facial tumors.^[7]^

Sporadic BL is distributed worldwide and is commonly observed in the United States and Europe.^[8]^ It has a weaker association with EBV infection, with only 20% to 30% of cases linked to EBV. Although it can occur at a wide range of ages, it is most frequently seen in children and adolescents.^[9]^ Immunodeficiency-associated BL primarily occurs in patients with AIDS or those in immunosuppressive states, where the correlation with EBV infection is higher. Unlike other HIV-related lymphomas, HIV patients with BL tend to have relatively higher CD4 counts (>200 cells/μL).^[8]^ Although Burkitt lymphoma can occur across a wide age range, its epidemiological patterns differ substantially by age. Endemic BL, which accounts for a large proportion of the global pediatric burden, predominantly affects young children, with a peak incidence between 4 and 7 years of age. Therefore, focusing on children under 10 years allows a more specific characterization of the early-childhood BL burden, which is closely linked to malaria exposure, early Epstein–Barr virus infection, and limited healthcare access in low-SDI regions.

The exact global incidence of BL in children under 10 years of age remains unclear due to limited resources in high-incidence regions, where the scarcity of diagnostic resources significantly hampers epidemiological data collection for BL, thus restricting effective management and prevention efforts.^[5]^ The 2021 Global Burden of Disease (GBD) study offers a comprehensive framework to assess the burden of BL across 204 countries and regions.^[10-12]^ In this study, we utilized data from the GBD 2021 to systematically describe BL in children under 10 years of age disease burden, providing global, regional, and national insights as well as multi-dimensional trend analyses. This research aims to assist healthcare providers and policymakers in developing relevant policies and optimizing resource allocation for improved BL management.

## 2. Materials and methods

### 2.1. Data sources

The present study drew upon data from the Global Burden of Disease (GBD) 2021 project, accessible through the Global Health Data Exchange platform (https://ghdx.healthdata.org/gbd-2021/sources),^[10]^ to assess the global burden of Burkitt lymphoma (BL) in children younger than 10 years. We quantified disease burden by estimating prevalence, incidence, mortality, and disability-adjusted life years (DALYs), and conducted stratified analyses across different Sociodemographic Index (SDI) categories.^[13,14]^ Following the GBD framework, countries and territories were aggregated into 21 regions based on geographic proximity and epidemiological characteristics. According to the International Classification of Diseases, 10th Revision (ICD-10), BL is coded as C83.7–C83.80, and in the GBD cause hierarchy, it is classified as a level 4 cause.^[10,11,15]^ Children under 10 years of age were selected a priori to specifically capture the burden of early-onset pediatric BL, which represents the predominant age group for endemic BL in high-burden regions. Missing or incomplete data were addressed within the GBD 2021 estimation framework. The GBD study applies standardized data processing procedures and Bayesian meta-regression models to integrate data from multiple sources and to generate estimates for locations and time periods with sparse or missing data. Uncertainty resulting from data limitations was quantified using 95% uncertainty intervals.

The GBD 2021 study integrates data from multiple sources to estimate the burden of Burkitt lymphoma, particularly in low-resource settings. These sources include population-based cancer registries, hospital discharge records, vital registration systems, and published epidemiological studies. In regions where direct data are sparse or incomplete, estimates are generated using Bayesian modeling approaches that borrow strength across neighboring locations, time periods, and related disease patterns. Cause-specific mortality and incidence estimates are produced using standardized GBD modeling pipelines, which ensure internal consistency across data sources and locations. Uncertainty arising from data sparsity is quantified and reported as 95% uncertainty intervals.

### 2.2. Descriptive analysis and trend analysis

In this analysis, we evaluated the global and regional burden of Burkitt lymphoma by calculating age-standardized rates (ASRs) and their 95% uncertainty intervals (UIs) for incidence, mortality, and disability-adjusted life years. From these estimates, we derived the age-standardized incidence rate (ASIR), prevalence rate (ASPR), mortality rate (ASMR), and DALY rate (ASDR). Temporal patterns from 1990 to 2021 were assessed using the estimated annual percentage change (EAPC).^[16]^ An EAPC with a 95% confidence interval (95% CI) lower bound above 0 was considered indicative of a significant increase, whereas a 95% CI upper bound below 0 denoted a significant decrease. Trends were regarded as stable when the 95% CI encompassed 0.

### 2.3. Decomposition analysis

To quantify the drivers of changes in BL-related DALYs from 1990 to 2021, we applied the standard GBD decomposition framework based on the Das Gupta method. Briefly, total changes in DALYs were decomposed into 3 additive components: population growth, changes in age structure, and epidemiological change.

This approach calculates counterfactual scenarios by sequentially substituting 1 component at a time while holding the others constant, and averages all possible decomposition orders to avoid path dependency.^[16,17]^

### 2.4. Cross-country inequality analysis

To support universal health coverage and guide policy, planning, and practice aimed at reducing health disparities, we assessed absolute and relative health inequalities in Burkitt lymphoma among children under 10 years using the Slope Index of Inequality (SII) and the Concentration Index (CI), respectively.^[18,19]^ The SII was derived by regressing national DALY estimates against each country’s relative position on the SDI scale, defined by the midpoint of its cumulative population distribution based on SDI ranking. The CI was computed as the ratio of the area between the Lorenz curve – constructed from cumulative SDI rank and cumulative DALY values – and the line of equality.^[20]^

### 2.5. Predictive analysis

We projected the global incidence, prevalence, mortality, and DALYs of Burkitt lymphoma in children under 10 years through 2040 to estimate the future global disease burden. Future projections were conducted using the NORDPRED package in R, which is based on an age–period–cohort (APC) Poisson regression framework. The model estimates age-specific rates using observed data from 1990 to 2021 and extrapolates recent temporal trends to predict future burden. To reduce the risk of overestimation, a drift attenuation approach was applied, whereby the linear drift component was gradually reduced in successive prediction periods, following standard NORDPRED recommendations. Population projections from the GBD 2021 framework were incorporated to estimate future counts of incidence, prevalence, mortality, and DALYs. Model validity was assessed by visual inspection of fitted versus observed trends and by ensuring consistency with historical patterns and uncertainty intervals.^[21-23]^ All statistical analyses and visualizations were performed in R, with a significance threshold set at *P* < .05.

## 3. Results

### 3.1. Disease burden globally and in 5 SDI regions

**Figure 1.**
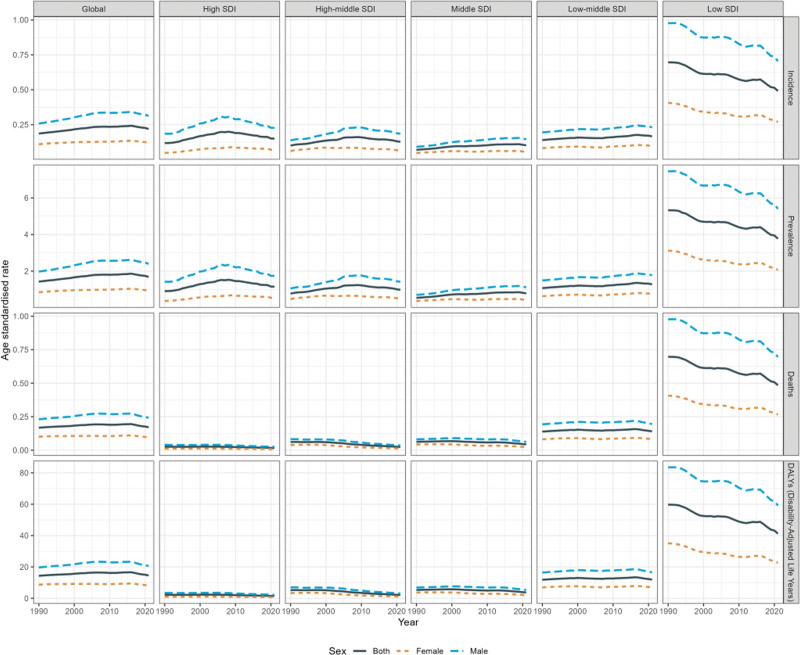
The ASIR, ASPR, ASMR and ASDR of BL among males and females globally and across the 5 SDI regions from 1990 to 2021. ASDR = age-standardized disability-adjusted life years rate, ASIR = age-standardized incidence rate, ASMR = age-standardized mortality rate, ASPR = age-standardized prevalence rate, ASR = age-standardized rates, BL = Burkitt lymphoma, DALY = disability-adjusted life years, SDI = Socio-Demographic Index.

In 2021, there were an estimated 22,878.70 (95% UI: 14,619.46–30,361.43) cases of BL in children under 10 years of age globally, with an age-standardized incidence rate (ASIR) of 0.22 (95% UI: 0.14–0.29) per 1,00,000 people, showing an upward trend since 1990, with an estimated annual percentage change (EAPC) of 0.72 (95% CI: 0.55–0.88). Notably, incidence, prevalence, mortality and DALYs for males were all significantly higher than for females. Among the 5 SDI regions, the low-SDI region had a substantially higher burden of BL compared to other areas, with a 2021 ASIR of 0.49 (95% UI: 0.28–0.68) per 1,00,000 and a more pronounced gender disparity in disease burden. Compared to 1990, only the low-SDI region showed a declining trend in BL burden in children under 10 years of age, with an EAPC of −0.94 (95% CI: −1.02 to −0.85) for incidence rates, while all other regions experienced an increase (Fig. 1, Tables S1 and S2, Supplemental Digital Content).

### 3.2. Burden of disease in 21 GBD regions

**Figure 2.**
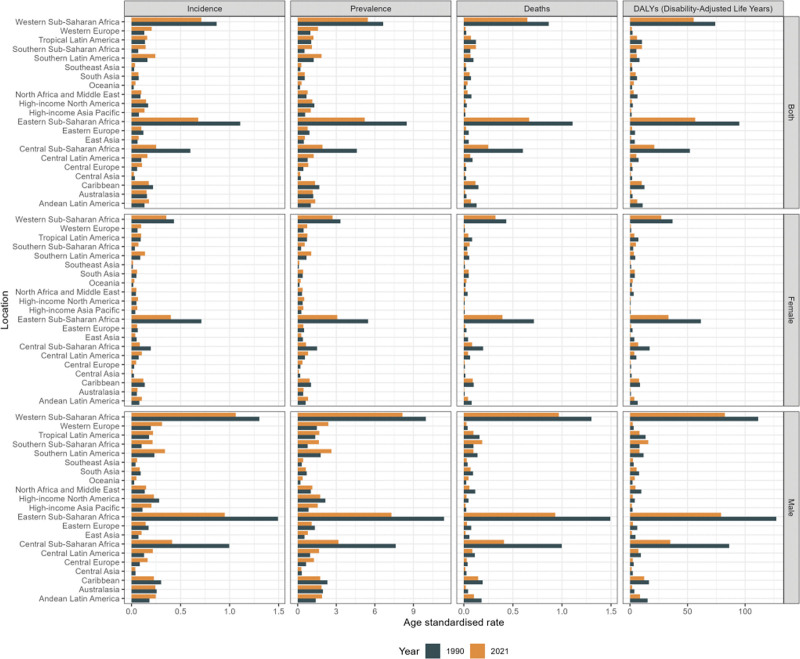
The ASIR, ASPR, ASMR and ASDR of BL among males and females across the 21 GBD regions in 1990 and 2021. ASDR = age-standardized disability-adjusted life years rate, ASIR = age-standardized incidence rate, ASMR = age-standardized mortality rate, ASPR = age-standardized prevalence rate, ASR = age-standardized rates, BL = Burkitt lymphoma, DALY = disability-adjusted life years, GBD = Global Burden of Disease.

In 2021, Western Sub-Saharan Africa and Eastern Sub-Saharan Africa had the highest incidence, prevalence, mortality and DALYs for BL in children under 10 years of age among the 21 GBD regions, with ASIRs of 0.71 (95% UI: 0.38–1.01) per 1,00,000 and 0.68 (95% UI: 0.39–0.99) per 1,00,000, respectively (Fig. 2 and Table S3, Supplemental Digital Content). Although these regions had higher incidence rates than others, both regions saw a decrease in incidence rates compared to 1990. Notably, Central Sub-Saharan Africa showed a significant reduction in BL burden in children under 10 years of age in 2021 compared to 1990, marking a substantial improvement in this area (Fig. 2 and Table S4, Supplemental Digital Content).

### 3.3. National disease burden in 2021

**Figure 3.**
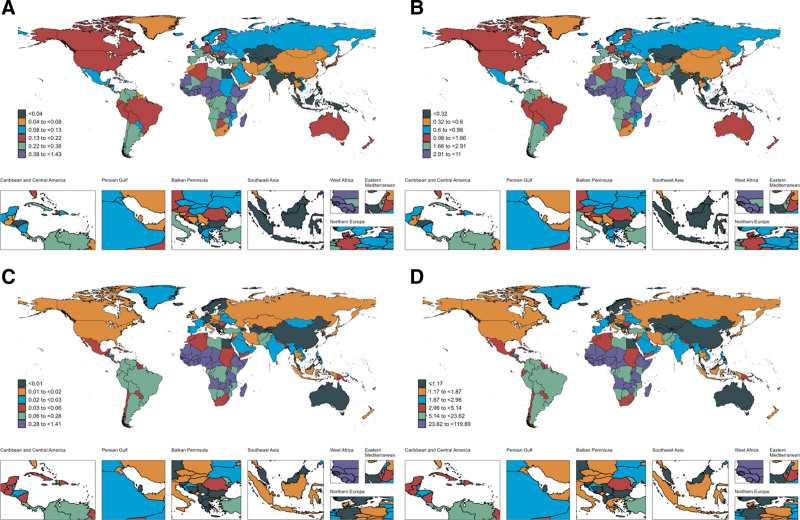
The ASIR, ASPR, ASMR and ASDR of BL in 204 countries in 2021. (A) ASIR; (B) ASPR; (C) ASMR; and (D) ASDR. ASDR = age-standardized disability-adjusted life years rate, ASIR = age-standardized incidence rate, ASMR = age-standardized mortality rate, ASPR = age-standardized prevalence rate, ASR = age-standardized rates, BL = Burkitt lymphoma.

In 2021, Nigeria had the highest number of BL cases in children under 10 years of age globally, with an estimated 4624.35 cases (95% UI: 2192.40–7001.90), followed by Uganda with 1519.29 cases (95% UI: 780.32–2663.15) and Ethiopia with 1268.53 cases (95% UI: 610.91–2142.46). Uganda recorded the highest incidence rate, with an ASIR of 1.43 per 1,00,000 (95% UI: 0.74–2.51), followed by Malawi at 1.41 per 1,00,000 (95% UI: 0.59–2.89) and South Sudan at 1.31 per 1,00,000 (95% UI: 0.54–2.35; Fig. 3 and Table S5, Supplemental Digital Content).

### 3.4. Decomposition analysis

**Figure 4.**
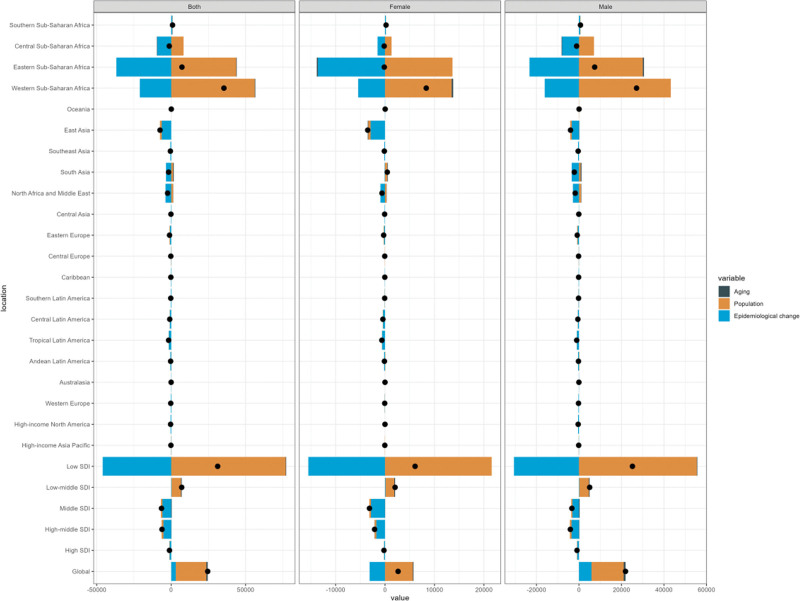
Changes in DALYs of BL according to aging, population growth and epidemiological change from 1990 to 2021 at global level by SDI quintile and by subgroups of sexes. The black dot denotes the overall value of the change resulting from all 3 components. For each component, the magnitude of a positive value suggests a corresponding increase in BL DALYs attributed to the component; the magnitude of a negative value suggests a corresponding decrease in BL DALYs attributed to the component. BL = Burkitt lymphoma, DALY = disability-adjusted life years, GBD = Global Burden of Disease, SDI = Socio-Demographic Index.

Decomposition analysis revealed the relative contributions of aging, population growth, and epidemiological change to the increase in BL-related DALYs across global regions (Fig. 4). Over the past 30 years, global DALYs for BL in children under 10 years of age have risen substantially, with the most significant increases observed in low SDI regions. Population growth emerged as the primary contributor to the global DALYs increase. Notably, epidemiological change showed different impacts by gender: it had a negative contribution to DALYs in females, whereas in males, it was the second largest contributor to the DALYs increase.

### 3.5. Inequality analysis

**Figure 5.**
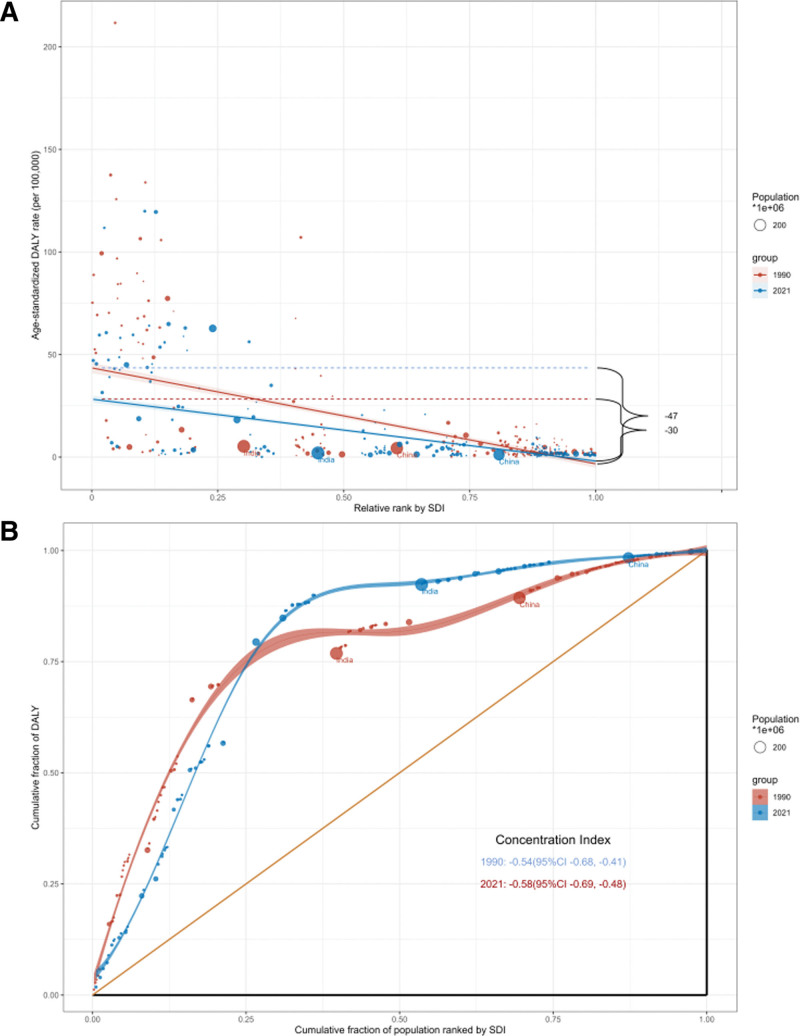
Health inequality slope index (A) and concentration index (B) for the DALYs of BL from 1990 to 2021 across the world. BL = Burkitt lymphoma, DALY = disability-adjusted life years, SDI = Socio-Demographic Index.

Inequality analysis of SDI-related BL burden in children under 10 years of age using the slope index indicated a decrease in the DALYs ratio disparity between the highest and lowest SDI countries, from −46.78 (95% CI: −51.94 to −41.62) in 1990 to −30.16 (95% CI: −33.71 to −26.61) in 2021 (Fig. 5). The concentration index rose from −0.54 (95% CI: −0.68 to −0.41) in 1990 to −0.58 (95% CI: −0.69 to −0.48) in 2021.

The decrease in the Slope Index of Inequality indicates a reduction in the absolute difference in BL-related DALYs between high- and low-SDI countries. However, the increase in the Concentration Index suggests that, relative to the overall global burden, BL remains increasingly concentrated in low-SDI countries.

This pattern reflects the fact that absolute reductions or slower increases in high-SDI settings do not necessarily translate into proportional improvements in low-SDI regions. As a result, relative inequality may worsen even when absolute differences narrow.

### 3.6. GBD future forecast for global BL

**Figure 6.**
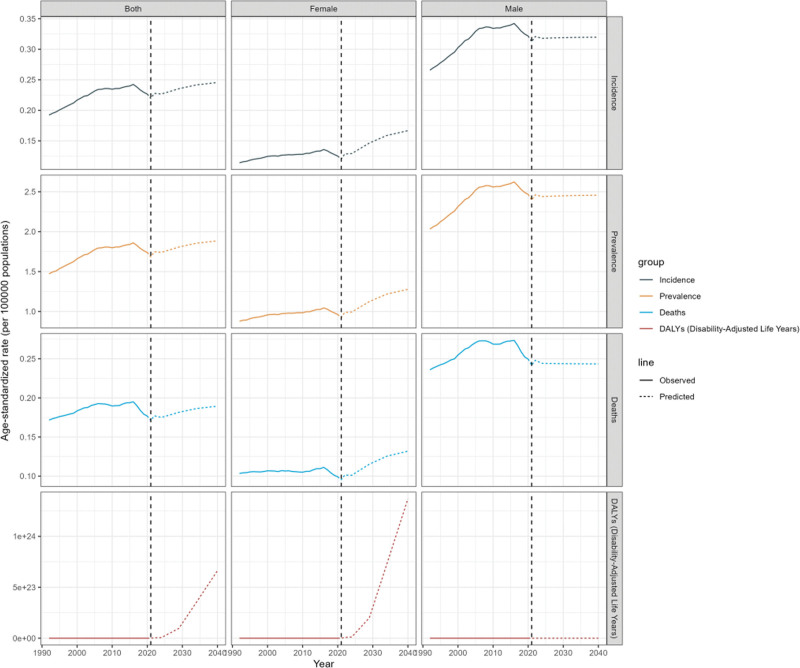
Future forecasts of GBD in BL. BL = Burkitt lymphoma, DALY = disability-adjusted life years, GBD = Global Burden of Disease.

Figure 6 shows future projections for BL in children under 10 years of age, with global ASIR, ASPR, ASMR and ASDR anticipated to gradually increase. By 2040, the global prevalence of BL in children under 10 years of age is projected to reach 23,755 cases, with approximately 2383 deaths and an ASIR of 0.25 per 1,00,000. In terms of gender, the burden of BL in children under 10 years of age is expected to increase primarily among females, who are estimated to account for around 7790 cases, about half of the projected cases for males. Although the absolute burden of BL remains higher among males, the projected rate of increase is relatively faster among females.

## 4. Discussion

This study systematically analyzed the global burden and epidemiological trends of BL in children under 10 years of age using GBD 2021 data, and projected future trends in BL burden in children under 10 years of age worldwide. Results indicate that the global incidence of BL in children under 10 years of age has shown an upward trend over the past 30 years, with notably higher ASIR and DALYs in low SDI regions compared to other areas. This research provides valuable epidemiological insights for public health policymakers to guide resource allocation and BL prevention efforts.

This study indicates that the global disease burden of BL in children under 10 years of age continues to rise, with notable sex differences observed in incidence, mortality, and DALYs. The BL burden in children under 10 years of age is significantly higher in males than females. Studies show that the incidence rate of sporadic BL in the United States is 2.6 times higher in males than in females.^[24]^ This disparity may relate to differences in immune responses, environmental exposures, and other biological factors between sexes. Research suggests that the risk across all types of BL may involve individual factors modulated by genetic or epigenetic regulators.^[25,26]^ Furthermore, sex-based differences in incidence underscore the influence of environmental factors like EBV and malaria.^[26]^ Importantly, studies have found that sex does not impact BL survival prognosis.^[27-30]^ Given the strong environmental determinants of BL, particularly malaria exposure and early Epstein–Barr virus infection, sex differences may reflect differential exposure patterns, healthcare access, or diagnostic practices rather than intrinsic biological susceptibility. In high-burden, low-resource settings, gender-related differences in health-seeking behavior, disease recognition, and reporting may further contribute to observed disparities.

There are significant differences in the burden of BL in children under 10 years of age across different SDI regions, with low SDI areas particularly affected. The burden of BL in children under 10 years of age in sub-Saharan Africa is notably higher than in other regions, closely linked to the high prevalence of malaria.^[31]^ This region’s elevated malaria burden is significantly associated with the risk of EBV infection in endemic BL, as malaria can increase the persistence of EBV infection through immunosuppressive mechanisms, thereby raising the incidence of BL.^[3,32,33]^ Additionally, the lack of adequate healthcare resources and diagnostic tools in low SDI areas further hampers effective control of BL. This study recommends enhancing preventive measures, such as malaria control and vaccination programs, in these high-burden areas to reduce the incidence of BL.

Decomposition analysis indicates that the global increase in DALYs for BL in children under 10 years of age is primarily driven by population growth, particularly in low SDI regions. Population growth and aging contribute to an increase in both the absolute number of BL cases and the overall burden, exacerbating the health challenges faced in low SDI areas.^[34-36]^ Gender-stratified analysis reveals that epidemiological changes have a negative contribution to the burden among females, suggesting that the increase in BL burden in children under 10 years of age for women is relatively slow, whereas it is the second-largest driving factor for DALYs growth in males. These findings suggest that future public health efforts should focus on targeted preventive measures for the male population and encourage low SDI regions to enhance basic healthcare infrastructure to alleviate the high burden of BL.

The burden of BL in children under 10 years of age in low SDI countries has exacerbated health inequality. Although there has been a reduction in absolute inequality for BL from 1990 to 2021, low SDI countries still bear a greater relative burden.^[37,38]^ Analysis using the slope index of health inequality and concentration index indicates that BL in children under 10 years of age is more concentrated in low SDI countries, highlighting significant challenges in the allocation of public health resources and policy formulation. We recommend increasing healthcare resources and implementing BL screening measures in low SDI regions to further reduce health inequalities associated with the burden of BL.

According to the predictive analysis of this study, the global burden of BL in children under 10 years of age is expected to continue rising in the future. Although males are projected to continue bearing a higher absolute burden of BL, the relatively faster increase observed among females suggests an emerging shift in the growth pattern of disease burden. This finding highlights the importance of incorporating sex-specific perspectives into future prevention and control strategies.

The findings suggest that prevention and control efforts for BL should prioritize gender factors and encourage future research to explore gender differences and other related biological mechanisms. This study’s predictions are based on global population growth data and existing trends for BL in children under 10 years. However, there remains some uncertainty regarding the accuracy of these predictions. This forecast provides a scientific basis for planning global BL prevention measures and ensuring the rational allocation of resources.

Several limitations of this study should be acknowledged. First, estimates of Burkitt lymphoma burden in low-resource settings rely partly on model-based approaches due to limited availability of pathology services, cancer registries, and immunophenotypic confirmation. In many high-burden regions, particularly in sub-Saharan Africa, definitive diagnosis of BL may be constrained by inadequate access to histopathology, cytogenetic testing, and EBV-related diagnostics, which may introduce uncertainty into incidence and mortality estimates. Second, diagnostic misclassification remains a potential concern. Burkitt lymphoma shares overlapping clinical and morphological features with other aggressive B-cell lymphomas, and in settings with limited diagnostic capacity, cases may be misclassified or grouped under broader lymphoma categories. Such misclassification could affect the precision of BL-specific burden estimates. Third, uncertainty related to disease coding should be considered. In the GBD framework, BL is identified using ICD-10 codes C83.7–C83.80. Variability in coding practices across countries and over time may contribute additional uncertainty, particularly in regions where cause-of-disease attribution is less standardized. Although the GBD study applies harmonized mapping and modeling procedures to improve comparability, residual uncertainty remains and is reflected in the reported uncertainty intervals. Despite these limitations, the standardized GBD methodology enables systematic comparisons across locations and time periods, providing valuable insights into broad epidemiological patterns and health inequalities associated with BL in children.

## 5. Conclusion

This study utilizes data from GBD 2021 to comprehensively analyze the global and regional disease burden of Burkitt lymphoma in children under 10 years, providing valuable epidemiological information for the improvement of public health policies. The impact of Burkitt lymphoma is significantly higher in low-SDI regions compared to others, highlighting the need for health policymakers to implement more effective measures to manage disease progression and improve the allocation of healthcare resources, thereby alleviating the burden of Burkitt lymphoma in these areas.

## Author contributions

**Conceptualization:** Siqi Zhang, Tian Xia, Xiong Wang.

**Data curation:** Siqi Zhang.

**Investigation:** Bin Pei.

**Methodology:** Siqi Zhang, Tian Xia, Yu Li.

**Software:** Siqi Zhang.

**Visualization:** Tian Xia, Guoxin Huang.

**Writing – original draft:** Siqi Zhang.

**Writing – review & editing:** Yu Li, Ren Niu, Guoxin Huang, Xiong Wang.










